# USP7 Inhibition Suppresses Neuroblastoma Growth via Induction of p53-Mediated Apoptosis and EZH2 and N-Myc Downregulation

**DOI:** 10.3390/ijms241813780

**Published:** 2023-09-07

**Authors:** Christophe Le Clorennec, Karen Lee, Yuchen Huo, Peter E. Zage

**Affiliations:** 1 Department of Pediatrics, Division of Hematology-Oncology, University of California San Diego, La Jolla, CA 92093, USA; 2 Peckham Center for Cancer and Blood Disorders, Rady Children’s Hospital, San Diego, CA 92123, USA

**Keywords:** neuroblastoma, USP7 inhibitor, MDM2, p53, deubiquitinase, ubiquitination, EZH2, N-Myc

## Abstract

Neuroblastoma (NB) is a pediatric malignancy originating from neural crest cells of the sympathetic nervous system that accounts for 15% of all pediatric cancer deaths. Despite advances in treatment, high-risk NB remains difficult to cure, highlighting the need for novel therapeutic approaches. Ubiquitin-specific protease 7 (USP7) is a deubiquitinase that plays a critical role in tumor suppression and DNA repair, and *USP7* overexpression has been associated with tumor aggressiveness in a variety of tumors, including NB. Therefore, USP7 is a potential therapeutic target for NB. The tumor suppressor p53 is a known target of USP7, and therefore reactivation of the p53 pathway may be an effective therapeutic strategy for NB treatment. We hypothesized that inhibition of USP7 would be effective against NB tumor growth. Using a novel USP7 inhibitor, Almac4, we have demonstrated significant antitumor activity, with significant decreases in both cell proliferation and cell viability in *TP53* wild-type NB cell lines. USP7 inhibition in NB cells activated the p53 pathway via USP7 and MDM2 degradation, leading to reduced p53 ubiquitination and increased p53 expression in all sensitive NB cells. In addition, USP7 inhibition led to decreased N-myc protein levels in both *MYCN*-amplified and -nonamplified NB cell lines, but no correlation was observed between *MYCN* amplification and treatment response. USP7 inhibition induced apoptosis in all *TP53* wild-type NB cell lines. USP7 inhibition also induced EZH2 ubiquitination and degradation. Lastly, the combination of USP7 and MDM2 inhibition showed enhanced efficacy. Our data suggests that USP7 inhibition may be a promising therapeutic strategy for children with high-risk and relapsed NB.

## 1. Introduction

Neuroblastoma (NB) is the most common extracranial solid tumor in children and the most common cancer during the first year of life [1]. NB patients have a broad range of clinical phenotypes, but children with high-risk NB have poor responses to treatment and high likelihood of relapse, with 5-year overall survival rates of 40–50% despite aggressive, multimodal therapy [2]. Therefore, novel treatments are needed for these children. 

The ubiquitin-proteasome system (UPS) plays an essential role in post-translational protein modification and serves many cellular functions through the selective degradation of regulatory proteins. The UPS also plays critical roles in protein trafficking, cell cycle progression, DNA damage repair, and chromatin remodeling [3]. Ubiquitination involves adding ubiquitin molecules to target proteins through ubiquitin ligase enzymes. The patterns of ubiquitination, including mono- or polyubiquitination, ultimately determine the fate of the target proteins. The UPS is further regulated by deubiquitinating enzymes (DUBs), which remove ubiquitin molecules from target proteins and protect them from degradation [4,5]. Dysregulation of the balance between ubiquitination and deubiquitination is linked to many human disorders, including cancer and neurodegenerative diseases [6,7]. 

Ubiquitin specific protease 7 (USP7) is a DUB that has been shown to play critical roles in regulating numerous proteins involved in tumor suppression, DNA repair, immune response, and epigenetic modulation [8]. Elevated *USP7* expression is directly correlated with tumor aggressiveness and poor prognosis for many tumors, including NB [9,10,11,12,13,14]. Proposed targets of USP7 implicated in NB include p53, MDM2, and N-myc. However, the full scope of the targets of USP7 and their potential roles in NB pathogenesis is unknown.

Almac4 is a highly potent and selective small-molecule inhibitor of USP7 that has shown significant antitumor activity in preclinical models of cancer [15]. Almac4 is a second generation USP7 inhibitor, along with GNE-6776, XL188, XL177A, FT671, and FT827, and these second generation inhibitors are thought to have fewer off-target activities compared to earlier generation USP7 inhibitors [16,17,18]. Specific pharmacologic inhibition of USP7 would allow us to study the direct effects of USP7 on its various targets and understand the functional role of USP7 in NB. We therefore hypothesized that USP7 inhibition with Almac4 would be effective against NB cells via modulation of ubiquitination of key proteins important in NB pathogenesis.

## 2. Results

### 2.1. High Expression of USP7 Is Associated with Worse Prognosis in Neuroblastoma

**Figure 1:**
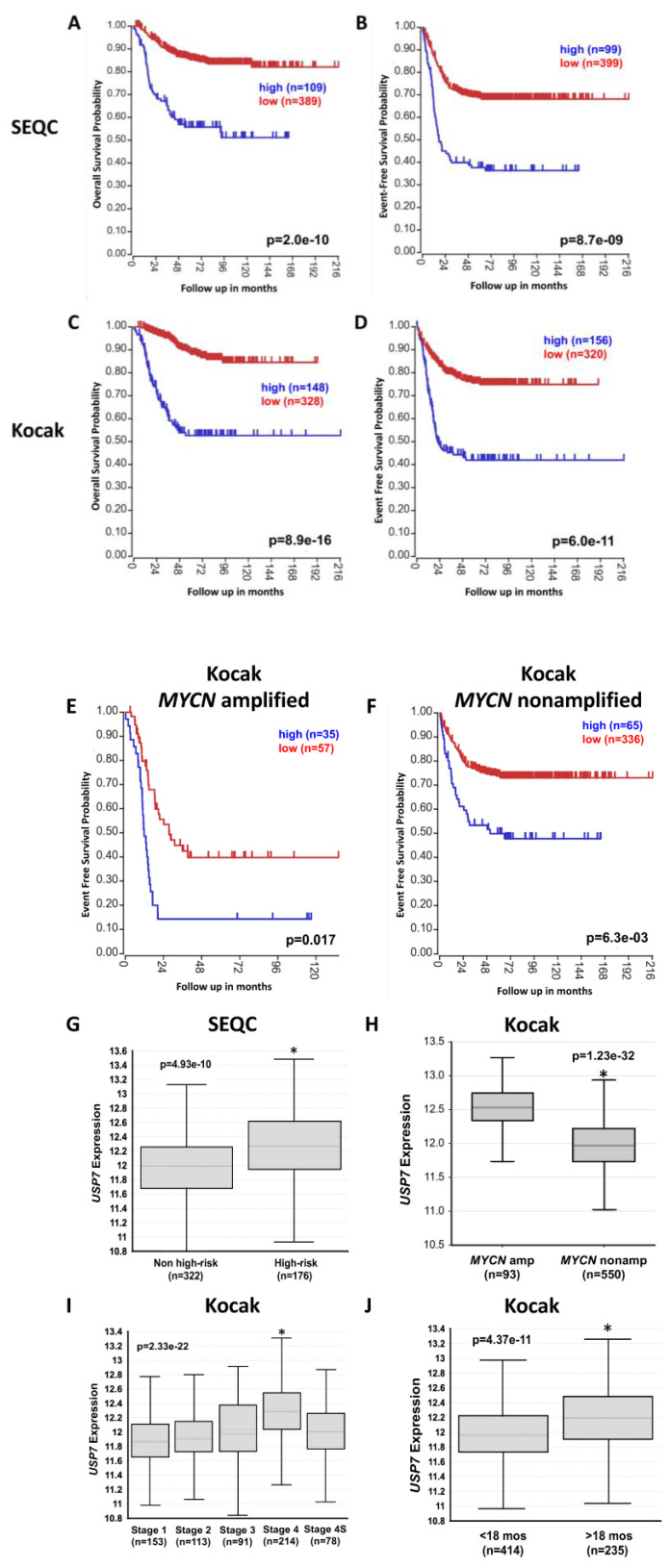
Neuroblastoma patient prognosis and outcomes impacted by *USP7* expression. Using the Kocak and SEQC datasets from R2 Genomics Analysis and Visualization Platform (http://r2.amc.nl; accessed on 8 May 2020): (**A**,**B**) Kaplan–Meier curves depicting overall survival (OS; **A**) and event-free survival (EFS; **B**) among all NB patients in the SEQC database, separated by tumors with high expression (in blue) vs. low expression (in red) of *USP7*, with patient numbers in parentheses. (**C**,**D**) Kaplan–Meier curves depicting OS (**C**) and EFS (**D**) among all NB patients in the Kocak database, separated by tumors with high expression (in blue) vs. low expression (in red) of *USP7*, with patient numbers in parentheses. (**E**,**F**) Kaplan–Meier curves depicting EFS among NB patients in the Kocak database with tumors with amplified *MYCN* (**E**) and non-amplified *MYCN* (**F**), separated by tumors with high expression (in blue) vs. low expression (in red) of *USP7*, with patient numbers in parentheses. (**G**) Relative *USP7* expression levels in tumors from the SEQC dataset were plotted in patients with high-risk and non-high-risk tumors (* *p* = 4.93 × 10^−10^). (**H**,**I**,**J**) Relative *USP7* expression levels in tumors from the Kocak dataset were plotted in patients with *MYCN*-amplified and -nonamplified tumors (* *p* = 1.23 × 10^−32^) (**H**), with stages 1, 2, 3, 4, and 4S tumors (* *p* = 2.33 × 10^−22^, stage 4 vs. stages 1,2,3,4S) (**I**) and in patients more or less than 18 months of age at diagnosis (* *p* = 4.37 × 10^−11^) (**J**).

To determine the associations of *USP7* expression with NB patient outcomes and prognostic features, we used NB patient tumor gene expression data from the R2 Genomics Analysis and Visualization Platform. Patients with high *USP7* gene expression had significantly worse overall and event-free survival rates compared to those with low *USP7* expression in the SEQC (Figure 1A,B) and Kocak (Figure 1C,D) datasets. Patients with high *USP7* expression also had lower survival rates, regardless of whether tumors had amplified and nonamplified *MYCN* (Figure 1E,F). *USP7* expression was also higher in tumors from patients with high-risk disease and in patient tumors with *MYCN* amplification (Figure 1G,H). *USP7* expression was also higher in tumors from patients with stage 4 disease and in tumors from patients older than 18 months of age at the time of diagnosis (Figure 1I,J). Taken together, these results support the association between higher *USP7* expression and worse prognosis in patients with NB.

### 2.2. USP7 Inhibition Decreases Cell Growth and Viability in TP53 Wild-Type Neuroblastoma Cells

**Figure 2:**
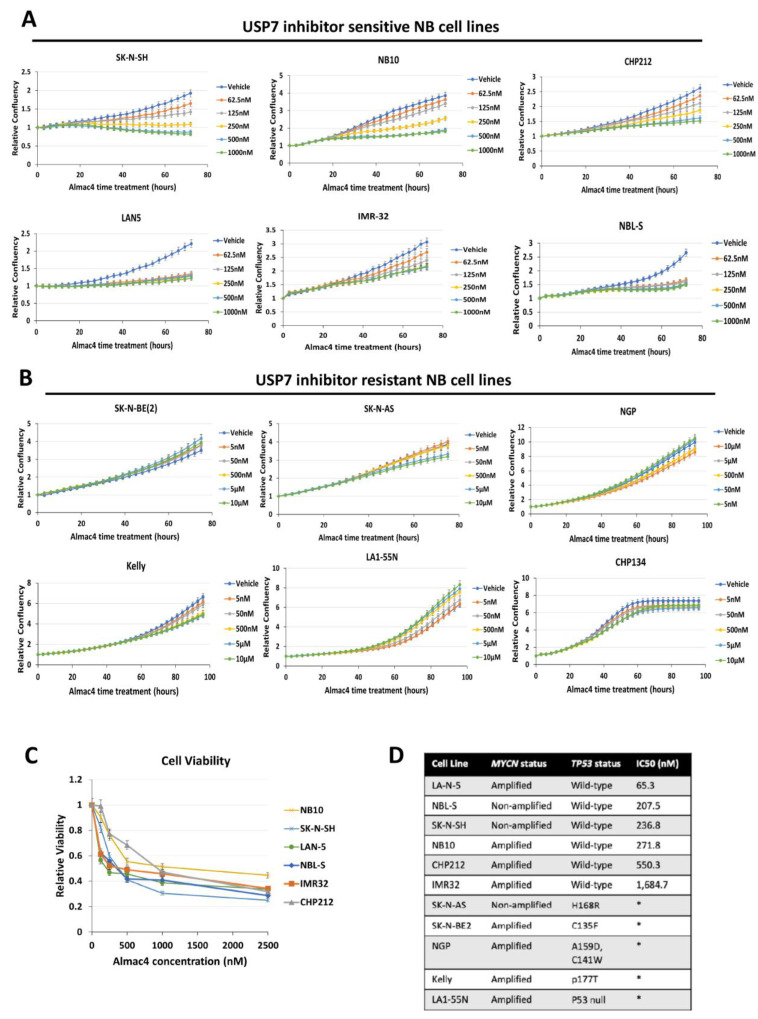
Efficacy of USP7 inhibition against neuroblastoma cell lines. (**A**,**B**) Relative cell confluence of Almac4-sensitive NB cell lines (**A**) SK-N-SH, NB-10, CHP-212, LAN-5, IMR-32, NBL-S compared to relative cell confluence of Almac4-resistant NB cell lines (**B**) SK-N-BE(2), SK-N-AS, NGP, Kelly, LA1-55N, CHP-134, that were each treated with DMSO control or increasing concentrations of Almac4 over time. Cell confluence was measured using continuous live-cell imaging over 72 h and normalized to control cell confluence. (**C**) Cell viability of sensitive NB cell lines determined by AlamarBlue assays performed after 72 h of treatment with Almac4 at increasing concentrations. (**D**) Molecular characteristics of all tested NB cell lines with calculated IC50 values from cell confluence curves shown in (**A**,**B**) above. *TP53* mutation information was derived from published literature [19]. * IC50 could not be determined due to inability to achieve less than 50% cell viability in concentrations up to 10 µM.

To determine the effects of pharmacologic inhibition of USP7 on NB cell proliferation, 12 NB cell lines were cultured with increasing concentrations of Almac4 or vehicle control. Treatment with Almac4 resulted in significant decreases in both cell confluence and cell viability *in vitro* in a subset of NB cells (Figure 2A,C,D). However, Almac4 had no effect on a different subset of NB cells (Figure 2B,D). While there was no association between *MYCN* amplification status and sensitivity to Almac4 (Figure 2D), all sensitive NB cell lines were *TP53* wild-type, whereas all resistant cell lines were *TP53* mutant or *TP53*-null, suggesting that p53 status is an important contributor to the efficacy of USP7 inhibition in NB cells and confirming prior results demonstrating the ability of USP7 inhibitors to induce p53-dependent apoptosis in *TP53* wild-type NB cells [12].

### 2.3. USP7 Inhibition Induces Cell Apoptosis in TP53 Wild-Type Neuroblastoma Cells

**Figure 3:**
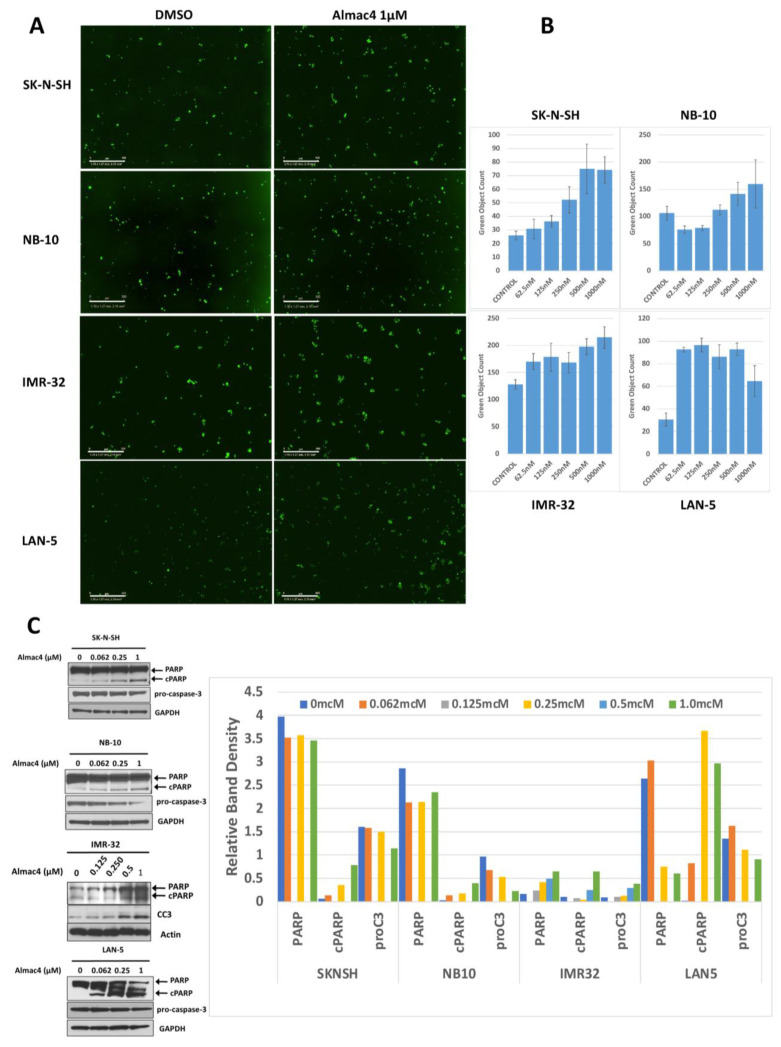
USP7 inhibition induces apoptosis in neuroblastoma cell lines. (**A**) SK-N-SH, NB-10, IMR-32, and LAN-5 NB cells were treated with DMSO control or 1 µM of Almac4 for 48 h and Caspase 3/7 reagent was added. Cells were monitored with continuous live cell imaging and total caspase cleavage was determined by identifying cells with activated caspase (green dots). Scale bars represent 300 µM (**B**) Fold change in total caspase count per image in SK-N-SH (*p* = 0.00011), NB-10 (*p* = 0.0022), IMR-32 (*p* = 0.0013) and LAN-5 (*p* = 2.47 × 10^−6^) NB cells treated with DMSO control or increasing concentrations of Almac4. (**C**) Lysates from SK-N-SH, NB-10, IMR-32, and LAN-5 NB cells treated with DMSO as control or with increasing concentrations of Almac4 were analyzed by Western blot for PARP cleavage and caspase-3 activation (as determined by decreasing procaspase-3 levels or increasing cleaved caspase-3 (CC3) levels). Band densitometry was performed using ImageJ and band densities for PARP, cleaved PARP (cPARP), and pro-caspase 3/CC3 (proC3) were normalized to control protein band densities (GAPDH or actin) and plotted.

To evaluate the mechanisms underlying the effects of USP7 inhibition on NB cell viability and confluence, we measured caspase-3/7 activation and PARP cleavage in sensitive NB cell lines treated with increasing concentrations of Almac4. Treatment with Almac4 resulted in increased caspase 3/7 cleavage (Figure 3A,B) and in increased PARP cleavage in a dose-dependent manner (Figure 3C), suggesting that USP7 inhibition by Almac4 induced the apoptotic machinery via caspase-3/7 activation.

### 2.4. USP7 Inhibition Destabilizes MDM2 and Stabilizes p53 in Neuroblastoma Cells

**Figure 4:**
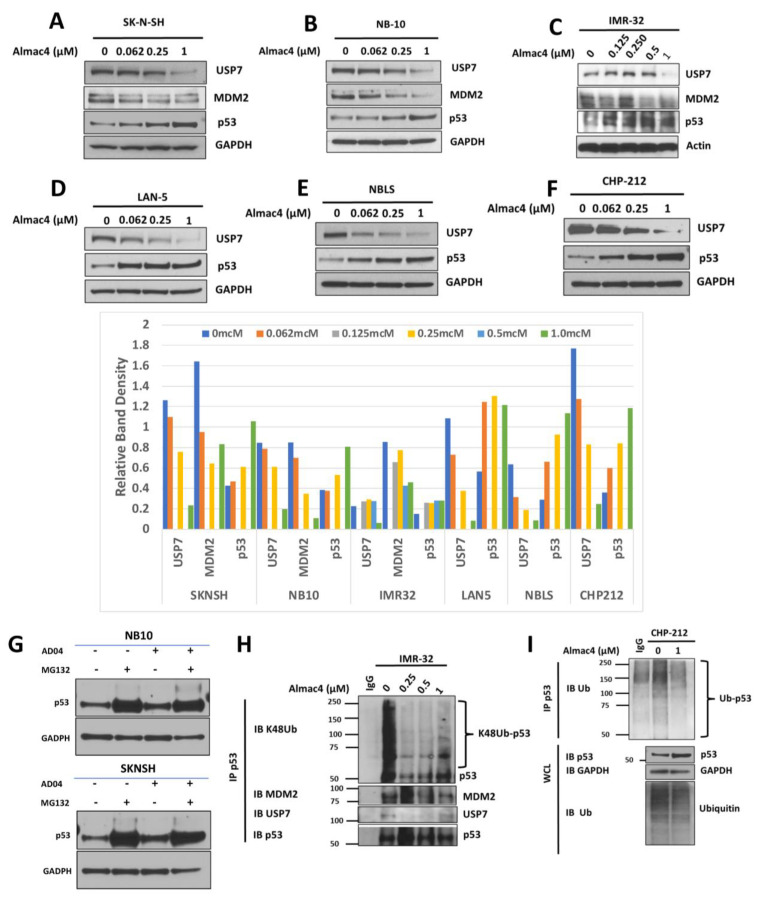
Impact of USP7 inhibition on expression and ubiquitination of MDM2 and p53. (**A**–**F**) Western blot analysis of USP7, MDM2, and p53 protein expression levels in SK-N-SH (**A**), NB-10 (**B**), IMR-32 (**C**), LAN-5 (**D**), NBL-S (**E**), and CHP-212 NB cells after treatment with DMSO or increasing concentrations of Almac4. Band densitometry was performed using ImageJ and band densities for USP7, MDM2, and p53 were normalized to control protein band densities (GAPDH or actin) and plotted. (**G**) Western blot analysis of p53 protein expression in SK-N-SH and NB-10 NB cell lines after treatment with DMSO or 1 µM Almac4 (AD04) alone or after pre-treatment with 5 µM MG132. (**H**) IMR-32 NB cells were treated with DMSO or increasing concentrations of Almac4, and immunoprecipitated p53 was analyzed by Western blot for lysine-48-linked (K48) ubiquitin, MDM2, USP7, and p53. (**I**) CHP-212 NB cells were treated with DMSO or with 1 µM of Almac4 and analyzed by Western blot for p53 and total ubiquitin (bottom panel) and immunoprecipitated p53 was analyzed by Western blot for total ubiquitin (top panel).

We then evaluated the effect of Almac4 on the protein levels of established USP7 targets. After treatment of the NB cell lines sensitive to Almac4, USP7 levels decreased in parallel to increasing Almac4 dose, suggesting that Almac4 induces USP7 degradation. In parallel to USP7 protein downregulation, a decrease in MDM2 protein levels was observed with concurrent increases in p53 protein levels in all tested NB cell lines (Figure 4A–F). To evaluate if p53 accumulation was due to inhibition of proteasomal degradation, we treated NB cells with Almac4 with and without proteasomal inhibitors. With the addition of a proteasome inhibitor, p53 protein levels were much higher for Almac4-treated NB cells (Figure 4G), confirming that the effect of Almac4 on p53 in NB cells was through its effect on the ubiquitin proteasome system. 

We then evaluated the pattern of ubiquitination of p53 in NB cells. At baseline, p53 is ubiquitinated on lysine-48 (K48) (Figure 4H). Almac4 treatment caused a significant reduction in p53 protein K48-ubiquitination in NB cells, suggesting that endogenous ubiquitin ligase activity was not sufficient to overcome USP7 inhibition by Almac4 (Figure 4H). In analogous studies in other NB cell lines, Almac4 treatment inhibited p53 ubiquitination and accumulation of p53 in treated cells (Figure 4I). 

### 2.5. USP7 Inhibition Destabilizes EZH2 in Neuroblastoma Cells

**Figure 5:**
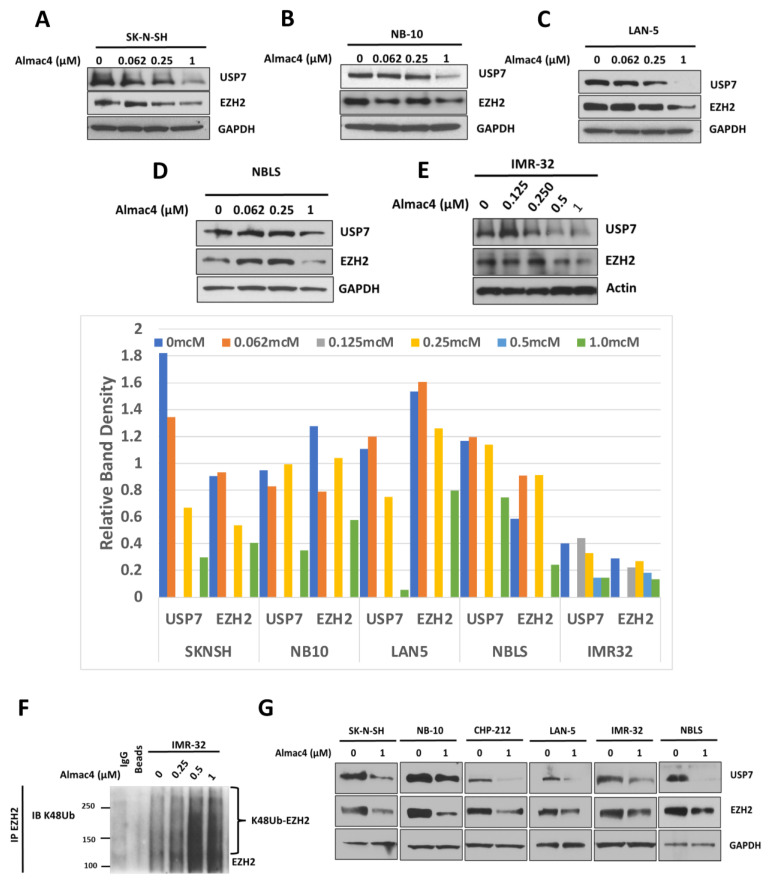
Impact of USP7 inhibition on expression and ubiquitination of EZH2. (**A**–**E**) Western blot analysis of USP7, EZH2, GAPDH, and actin protein expression levels in SK-N-SH (**A**), NB-10 (**B**), LAN-5 (**C**), NBL-S (**D**), and IMR-32 (**E**) NB cells after treatment with DMSO control or after increasing doses of Almac4. Band densitometry was performed using ImageJ and band densities for USP7 and EZH2 were normalized to control protein band densities (GAPDH or actin) and plotted (**F**) IMR-32 NB cells were treated with DMSO or increasing concentrations of Almac4, and immunoprecipitated EZH2 was analyzed by Western blot for lysine-48-linked (K48) ubiquitin. (**G**) Western blot analysis of USP7, EZH2, and GAPDH protein expression levels in SK-N-SH, NB-10, CHP-212, LAN-5, IMR-32, and NBL-S NB cells after treatment with DMSO or after 1 µM of Almac4 for 48 h.

Prior studies have reported that USP7 stabilizes EZH2 via deubiquitination in different types of adult cancers [15,20,21]. To determine the relationship between USP7 and EZH2 in NB cell lines, we evaluated the effect of Almac4 on EZH2 protein expression. Almac4 treatment led to decreased EZH2 protein expression in tested NB cells (Figure 5A–D,F,G). Increasing Almac4 doses also induced an increase in EZH2 K48-ubiquitination (Figure 5E), confirming a role for USP7 in the ubiquitination of EZH2 in NB. 

### 2.6. USP7 Depletion Decreases Neuroblastoma Cell Growth, Increases Protein Expression of p53, and Decreases Protein Expression of EZH2

**Figure 6:**
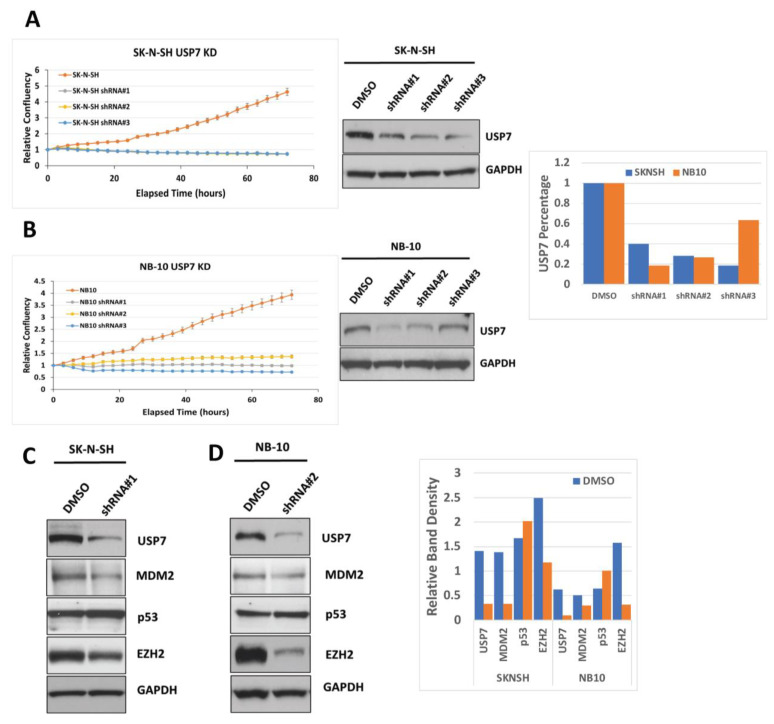
Impact of USP7 depletion on expression of MDM2, p53, and EZH2. (**A**,**B**) (Upper panels) Parental SK-N-SH (**A**) and NB-10 (**B**) NB cells and matching cell lines with USP7 depletion using 3 different shRNAs in each cell line were analyzed by live cell imaging for cell confluence over time. Relative cell confluence over 72 h is shown. (Lower panels) Western blot analyses of USP7 protein expression levels in matching NB cell lines from above. Band densitometry was performed using ImageJ and band densities for USP7 were normalized to control protein band densities (GAPDH) and plotted. (**C**,**D**) Western blot analysis of USP7, MDM2, p53, EZH2, and GAPDH protein expression levels in SK-N-SH (**C**) and NB-10 (**D**) parental and USP7 knockdown cell lines. Band densitometry was performed using ImageJ and band densities for USP7, MDM2, p53, and EZH2 were normalized to control protein band densities (GAPDH) and plotted.

To further evaluate the roles of USP7 expression on NB cell growth, we established stable *USP7* knockdown (KD) NB cell lines using three different *USP7* shRNA plasmids. USP7 protein expression was significantly reduced in USP7 KD cell lines compared to the parental cell lines (Figure 6A,B). USP7 depletion also led to significantly reduced cell growth compared to the parental cell lines (Figure 6A,B). In addition, USP7 depletion led to decreased expression of both MDM2 and EZH2 and an increase in p53 protein expression (Figure 6C,D), similar to that seen with pharmacologic inhibition of USP7 and further supporting a role for USP7 in NB tumor growth via to its effect on USP7 deubiquitinase activity, allowing for target protein degradation.

### 2.7. USP7 Inhibition and USP7 Depletion Leads to Decreased N-Myc Protein Expression

**Figure 7:**
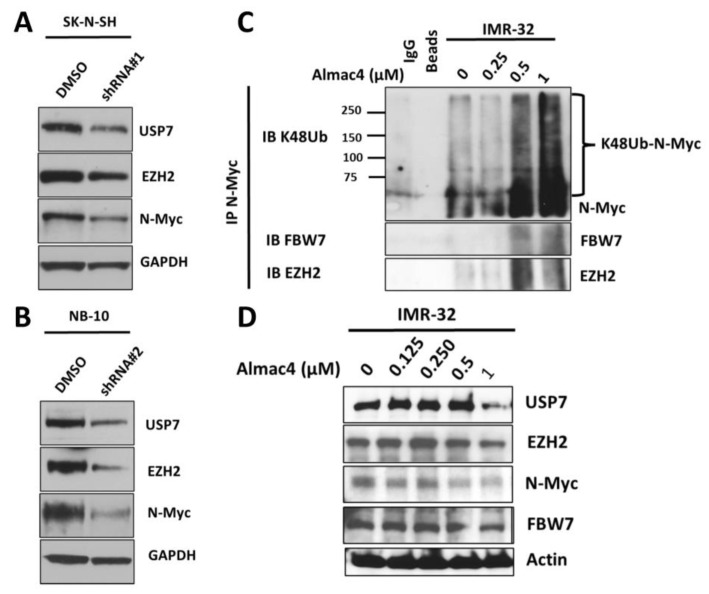
Impact of USP7 inhibition and depletion on expression and ubiquitination of N-myc. (**A**,**B**) Western blot analysis of USP7, EZH2 N-Myc, and GAPDH protein expression levels in SK-N-SH (**A**) and NB-10 (**B**) parental and *USP7* knockdown NB cell lines. (**C**) IMR-32 NB cells were treated with DMSO or increasing concentrations of Almac4, and immunoprecipitated N-myc was analyzed by Western blot for lysine-48-linked (K48) ubiquitin, FBW7, and EZH2. (**D**) Cell lysates of IMR-32 NB cells treated with DMSO or increasing concentrations of Almac4 were analyzed by Western blot for USP7, EZH2, FBW7, N-Myc, and actin protein expression levels.

Prior studies have reported that N-myc binds to EZH2, allowing for N-myc stabilization by counteracting the binding of the E3 ubiquitin ligase FBW7 to N-Myc, inhibiting N-Myc K48-ubiquitination and blocking N-Myc proteasomal degradation [22]. We therefore evaluated the change in N-myc protein expression with USP7 inhibition and depletion. After Almac4 treatment, N-myc protein expression decreased in both *MYCN*-amplified and *MYCN*-nonamplified NB cell lines (Figure 7D), with concurrent decreases in EZH2 levels (Figure 5A–D,F,G). USP7 depletion similarly resulted in decreased N-myc and EZH2 expression (Figure 7A,B), confirming the correlation between USP7 downregulation and EZH2 and N-Myc downregulation. Furthermore, with higher doses of Almac4, FBW7 co-immunoprecipitated with N-Myc, which was K48-ubiquitinated (Figure 7C). These results, taken together, confirm the correlation between EZH2 downregulation and N-Myc K48-ubiquitination by FBW7 in the context of USP7 inhibition. 

### 2.8. Combination of USP7 Inhibition and MDM2 Inhibition Leads to Enhanced Efficacy in Neuroblastoma Cells

**Figure 8:**
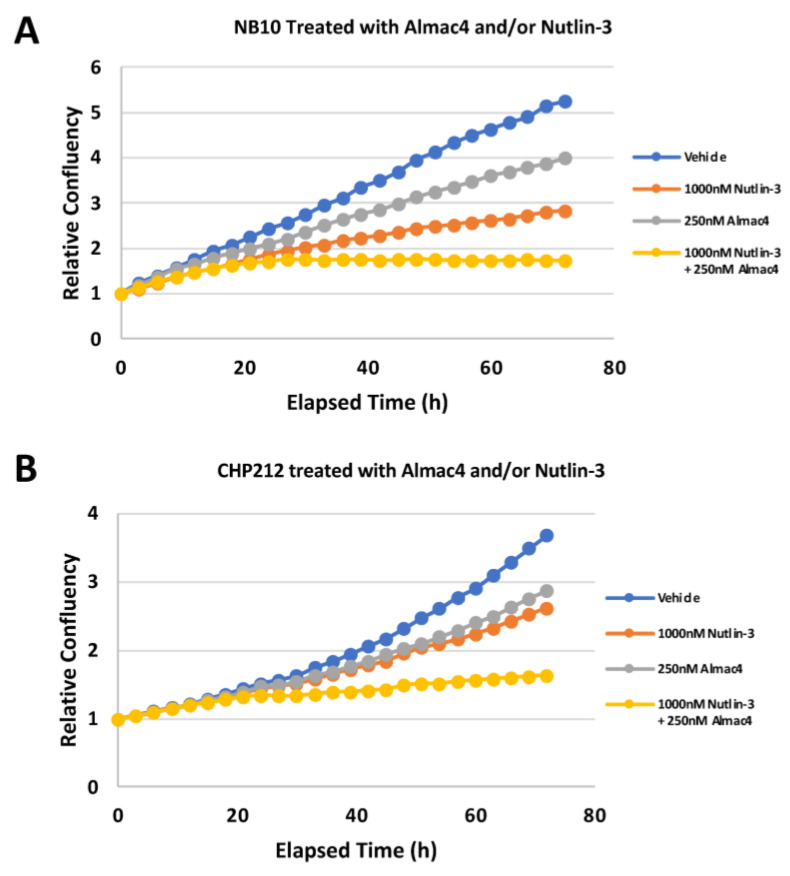
Relative efficacy of combined USP7 and MDM2 inhibition against neuroblastoma cell lines. (**A**,**B**) Relative cell confluence of NB10 (**A**) and CHP-212 (**B**) NB cells treated with DMSO, 250 nM Almac4, 1 µM Nutlin-3, or the combination of Almac4 and Nutlin-3. Cell confluence was measured using continuous live-cell imaging over 72 h of treatment.

Vertical inhibition (simultaneous inhibition of two targets along the same molecular pathway) has been shown to be an effective way to counteract development of resistance and improve survival [23]. To evaluate the efficacy of vertical inhibition along the USP7-MDM2–p53 pathway in NB cells, we studied the efficacy of treatment with Almac4 combined wit- MDM2 inhibition with Nutlin-3 in *MYCN*-amplified, *TP53* wild-type NB cell lines sensitive to Almac4 treatment. Combination treatment with Almac4 and Nutlin-3 resulted in significantly decreased cell viability compared to treatment with either Almac4 or Nutlin-3 alone at equivalent concentrations (Figure 8A,B). These results support the conclusion that targeting USP7 is a promising strategy for treating children with high-risk NB. 

## 3. Discussion

**Figure 9:**
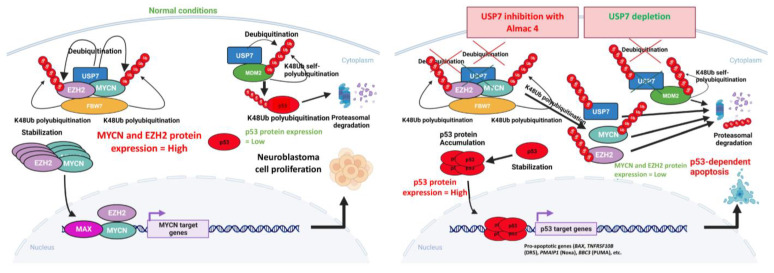
Model for the mechanisms of action of USP7 inhibition and USP7 depletion in neuroblastoma cells with wild-type p53.

Ubiquitin-specific protease 7 (USP7) is a deubiquitinase that plays a critical role in tumor suppression and DNA repair, and *USP7* overexpression has been associated with tumor aggressiveness in a variety of tumors, including NB. We have demonstrated that high expression of *USP7* is associated with poor prognosis in children with NB and with other biological features of poor prognosis such as *MYCN* amplification and increased tumor stage. We have also demonstrated that the novel USP7 inhibitor Almac4 is effective against NB tumor growth in *TP53* wild-type NB cells regardless of *MYCN* status through induction of caspase-mediated apoptosis, but Almac4 was less effective in *TP53*-mutated or *TP53*-null NB cells. We confirmed that Almac4 works through inhibition of USP7 and its effect on maintaining protein stabilization of various target proteins. Almac4 treatment and USP7 depletion led to decreased USP7, MDM2, N-myc, and EZH2 expression levels and to increased p53 expression, with reduced p53 ubiquitination and increased EZH2 ubiquitination (Figure 9). Vertical inhibition in the same pathway by inhibition of USP7 and MDM2 together also showed enhanced efficacy. Most importantly, we demonstrated that USP7 inhibition and depletion both destabilized EZH2, establishing a relationship between USP7 and EZH2 in NB for the first time. Our study provides biological and clinical rationale for additional preclinical testing of USP7 inhibitors in children with high-risk and relapsed NB and emphasizes the need for further research to characterize USP7′s various targets and understand the roles they play in NB tumor development and growth. 

USP7 has been shown to play an important role in the development, progression, and therapeutic response of multiple cancer types. Increased USP7 protein expression is directly related to tumor invasion in prostate cancer [24] and plays a major role in tumorigenesis of non-small cell lung carcinoma (NSCLCs) via p53-dependent pathways [25]. Other studies have demonstrated that changes in USP7 protein expression can modulate colon carcinoma growth and sensitivity to apoptosis in vivo via the stabilization of p53, due to USP7-mediated deubiquitination of p53 proteins [26,27,28], leading to high levels of p53 and restoring the sensitivity to apoptosis by irradiation [29]. USP7 can also maintain DNA damage response and promote cervical cancer [30], and high *USP7* expression is positively correlated with poor survival in patients with cervical cancer [30]. USP7 is frequently overexpressed in NB cell lines and patient tumors and has been associated with aggressive tumor behavior and poor clinical outcomes, further suggesting that USP7 inhibitors are likely to be effective against NB cells and tumors. 

Pharmacological inhibition of USP7 has been achieved during the last decade, and multiple novel agents (HBX42108, HBX19818, HBX28258, P5091, and P22077) have been reported to inhibit USP7 in cancer cells [8,31,32,33,34]. The first USP7 inhibitor, P5091, was previously shown to inhibit multiple myeloma proliferation [31]. Prior reports also demonstrated the efficacy of the USP7 inhibitor P22077 in NB models, where P22077 could efficiently induce p53-mediated apoptosis in NB cells with a functional p53 pathway and could inhibit NB growth in vivo [12]. In fact, one of the molecular features that distinguishes NB from the majority of adult malignancies is that most NB tumors have a wild-type *TP53* gene with a functional p53 pathway, rendering most NB tumors sensitive to the reactivation of the p53-dependent apoptotic downstream pathway [35,36,37]. Prior studies have also shown that the USP7 inhibitors P5091 and P22077 inhibit NB cell growth through destabilization of N-Myc, independent of *TP53* status, and identified direct interactions between USP7 and N-Myc [13]. Our results obtained with the USP7 inhibitor Almac4 in NB cell lines have confirmed these prior results with other USP7 inhibitors on NB cell lines. We observed the same effects on NB cells with wild-type *TP53* with lower concentrations (approximately 10-fold less than with P22077 [12]). 

Increased N-Myc expression has been shown to repress and alter p53 target gene expression directly, inhibiting p53 activation response to DNA damage, apoptosis, and cell cycle arrest and potentially explaining the resistance to chemotherapy observed in *MYCN*-amplified tumors [38]. In addition, USP7 inhibition was reported to restore chemosensitivity in *MYCN*-overexpressing small-cell lung cancer models [39]. The significance of these interactions of USP7 with N-myc and p53 in NB tumorigenesis and chemoresistance is not well understood and requires further clarification. Targeting MDM2 directly by specific inhibitors has been shown to be able to reactivate p53 [40,41]. USP7 stabilizes MDM2 by deubiquitination, allowing MDM2 to ubiquitinate p53, leading to proteasomal degradation of p53. Inhibition of USP7 deubiquitinase function therefore promotes increased MDM2 ubiquitination, leading to MDM2 degradation and resulting in reduced p53 protein degradation and subsequent accumulation and activation. The specific MDM2 inhibitor, Nutlin-3, which blocks the interaction of p53 with MDM2, has been shown to sensitize chemotherapy-induced apoptosis in NB by reactivation of the p53 pathway [31,42,43,44]. In this study, we have shown that inhibition of USP7 by Almac4 led to increased p53 expression in NB cells, likely via destabilization of MDM2. We also demonstrated that the combination of an MDM2 inhibitor, Nutlin-3, with Almac4, resulted in increased efficacy, confirming that vertical inhibition of the same pathway by inhibition of both the USP7 deubiquitinase in addition to the inhibition of the MDM2-p53 interaction demonstrates enhanced efficacy compared to either inhibitor alone. 

Enhancer of zeste homolog 2 (EZH2), which is a member of the PRC2 complex and acts as a methyltransferase, regulates genes involved in tumorigenesis by silencing gene expression via methylation of lysine 27 of the histone H3. High EZH2 protein expression has been shown to be correlated with unfavorable prognosis in NB, and inhibition of EZH2 methyltransferase function or downregulation of EZH2 protein expression by EZH2 shRNA knockdown were both shown to inhibit NB growth and differentiation [45,46]. N-myc was shown to interact directly with EZH2, leading to suppression of its target genes in NB and in neuroendocrine prostate cancer cells [47,48]. Recently, EZH2 was found to be a target substrate for USP7, and USP7 deubiquitinates and stabilizes EZH2 and promotes cell migration, invasion, and sphere-forming potential in prostate and colon cancer cells. Knockdown of USP7 further led to decreased EZH2 and growth inhibition [20,21,49]. While the relationship between USP7 and EZH2 and its significance in NB pathogenesis have not yet been established, our results have shown direct correlations between USP7 downregulation induced by inhibition of its deubiquitinase activity via Almac4 or by USP7 depletion and EZH2 and N-Myc ubiquitination and protein degradation. Recent studies have reported that EZH2 can interact directly with N-Myc and c-Myc, promoting their stabilization independently of EZH2 methyltransferase function by competing for direct Myc binding with the SCF-FBXW7 E3 ubiquitin ligase complex and inhibiting their ubiquitination by FBXW7 [22]. Depletion of EZH2 led to N-Myc polyubiquitination by SCF-FBXW7, and subsequent N-Myc degradation reduced tumor cell growth in *MYCN*-amplified NB and small cell lung carcinoma cells [22]. Our results confirm that both USP7 inhibition and depletion induced K48-specific polyubiquitination of EZH2 and N-Myc, and we confirmed SCF-FBXW7 interactions with N-Myc and EZH2 after Almac4 treatment, resulting in both EZH2 and N-myc degradation.

In summary, we have demonstrated significant antitumor activity of Almac4, a novel USP7 inhibitor, against NB cells, with significant decreases in both cell proliferation and cell viability in p53 wild-type NB cell lines. USP7 inhibition in NB cells activated the p53 pathway via USP7 and MDM2 degradation, leading to reduced p53 ubiquitination and increased p53 expression in all sensitive treated NB cells. In addition, USP7 inhibition led to decreased N-myc protein levels in both *MYCN*-amplified and -nonamplified NB cell lines, but no correlation was observed between *MYCN* amplification and treatment response. USP7 inhibition induced apoptosis in all *TP53* wild-type NB cell lines. USP7 inhibition also induced EZH2 ubiquitination and degradation. Lastly, the combination of USP7 and MDM2 inhibition showed enhanced efficacy. Our data suggests that USP7 inhibition may be a promising therapeutic strategy for children with high-risk and relapsed NB.

## 4. Materials and Methods

### 4.1. Cell Lines and Cell Culture

NB cell lines (SK-N-AS, SK-N-BE(2), SK-N-SH, SJ-NB-10, NBL-S, CHP-212, IMR-32, NGP, Kelly, LAN-5, LA1-55N, CHP-134) used in this study have been previously utilized by our laboratory [50,51,52] and were purchased from American Type Culture Collection (ATCC, www.atcc.org) or were generously provided by Susan Cohn (The University of Chicago Children’s Hospital, Chicago, IL, USA) or John Maris (Children’s Hospital of Philadelphia, Philadelphia, PA, USA). NB cells were maintained in RPMI-1640 media (Corning Life Science, Corning, NY, USA) supplemented with 10% heat-inactivated fetal bovine serum (FBS) (Life Technologies, Grand Island, NY, USA), 1% L-glutamine, 1% sodium pyruvate, and 1% non-essential amino acids and antibiotic/antimycotic solution (Sigma-Aldrich, St. Louis, MO, USA) at 37 °C in 5% CO_2_. 

### 4.2. Reagents and Antibodies

Almac4 was generously provided by Almac Group (Craigavon, UK). Nutlin-3 (444151) and MG132 (474791) were purchased from Sigma-Aldrich (St. Louis, MO, USA). DMSO (20688) was purchased from Thermo Scientific (Waltham, MA, USA). Rabbit monoclonal antibodies anti-EZH2 (5246S), anti-HAUSP (4833S), anti-p53 (2527S), anti-GAPDH (5174S), anti-vinculin (13901S), and rabbit polyclonal antibodies anti-PARP (9542S), anti-caspase-3 (9262S), anti-cleaved caspase-3 (9661S), anti-ubiquitin (3933S), anti-K48-linkage polyubiquitin (8081S), and mouse monoclonal antibody anti-p53 (18032S) were purchased from Cell Signaling Technology (Danvers, MA, USA). Mouse monoclonal anti-MDM2 (clone SMP14, sc965) antibody was purchased from Santa Cruz Biotechnology (Dallas, TX, USA). Mouse monoclonal anti-N-myc (OP13) and anti-β-actin (AS316) antibodies were purchased from Sigma-Aldrich (St. Louis, MO, USA). Rabbit polyclonal anti-FBWX7 (ab192328) antibody was purchased from Abcam (Waltham, MA, USA). Goat anti-rabbit IgG (H+L)-HRP secondary antibody (1706515) and goat anti-mouse IgG (H+L)-HRP secondary antibody (1706516) were purchased from Bio-Rad (Hercules, CA, USA). 

### 4.3. Transfection

Short hairpin plasmids for USP7 TRCN0000004058 (shRNA #1), TRCN0000004059 (shRNA #2), and TRCN0000318578 (shRNA #3) were purchased from MISSION shRNA (Sigma-Aldrich, St. Louis, MO, USA). NB cells were cultured in 6-well plates and incubated at 37 °C. Cells were transfected using Polyplus jetOPTIMUS DNA transfection reagent using 1 µg plasmid DNA. Puromycin (1 µg/mL for NB-10, 2 µg/mL for SK-N-SH) was added to select transfected cells and to maintain a pressure of selection during cell growth and future passage of transfected NB cells. 

### 4.4. Cell Confluence Assay

NB cells were plated in 96-well plates at a seeding density of 1 × 10^5^ cells/well and incubated at 37 °C. For drug treatment experiments, increasing concentrations of Almac4 alone, Nutlin-3 alone, combinations of Almac4 and Nutlin-3, or equivalent doses of DMSO were added to the wells. The plates were placed into the Incucyte ZOOM^TM^ Live Cell Analysis System. Phase contrast images were taken every 3 h for up to 96 h, and cell growth over time was quantified using percent cell confluence normalized to DMSO control. For shRNA experiments, cell growth over time was quantified using percent cell confluence normalized to parental cell line control. 

### 4.5. Cell Viability Assay

NB cells were plated in 96-well plates at a seeding density of 1 × 10^5^ cells/well and incubated at 37 °C. Increasing concentrations of Almac4 or equivalent doses of DMSO were added to the wells. After 72 h of treatment, alamarBlue™ Cell Viability Reagent (Invitrogen, Waltham, MA, USA) was added to each well at 10% of total volume. Cells were then incubated at 37 °C for 1–5 h, and fluorescence was measured using a microplate reader at 560 nm (excitation) and 590 nm (emission). Viability was normalized to control wells containing only media. IC50 concentrations were calculated by using the online Quest Graph™ IC50 Calculator (AAT Bioquest, Inc., Pleasanton, CA, USA) [28]. 

### 4.6. Caspase 3/7 Apoptosis Assay

NB cells were plated in 96-well plates at a seeding density of 1 × 10^5^ cells/well and incubated at 37 °C. Cells were then treated with increasing concentrations of Almac4 or equivalent doses of DMSO along with the Incucyte Caspase-3/7 Green Dye for Apoptosis (Essen BioScience, Ann Arbor, MI, USA) at 1:1000 concentration. Phase contrast and green fluorescence images were taken every 3 h using the Incucyte Zoom^TM^ live cell imaging system. Green fluorescence images and the green object count per image were used for analysis. Statistical significance of observed changes in green object count was determined using single-factor ANOVA.

### 4.7. Western Blotting

NB cells were plated in 6-well plates or in 10 cm culture dishes and incubated overnight at 37 °C. For drug treatment experiments, cells were cultured with increasing concentrations of Almac4 or equivalent concentrations of DMSO for 18–24 h. For studies with MG132, cells were cultured with 5 µM MG132 for 2 h followed by 1000 nM Almac4 or equivalent concentrations of DMSO for 6 h. Cells were then washed with cold PBS and lysed with cold lysis buffer containing RIPA buffer (50 mM Tris pH7.5, 150 mM NaCl, 2 mM EDTA pH8, 0.5% deoxycholic acid, 0.1% SDS, 1% Triton X-100, qsp H_2_O) with added protease and phosphatase inhibitor tablets (Thermo Scientific, Waltham, MA, USA). Protein concentrations were determined using the Pierce BCA Protein Assay Kit (Thermo Scientific, Waltham, MA, USA) and absorbance was measured using a microplate reader at 562 nm. 1 mg protein lysate was directly mixed with 2X SDS Laemmli buffer (1 mM TRIS pH 6.8, 1% SDS, 40% glycerol, 0.1% bromophenol blue, 20% β-Mercaptoethanol) and heated at 95 °C for 10 min before electrophoresis. Equal amounts of protein (30 μg) were loaded and separated via sodium dodecyl sulfate polyacrylamide gel electrophoresis using Bolt 4–12% Bis-Tris Plus precast gels (Invitrogen, Waltham, MA, USA) and transferred to polyvinylidene fluoride (PVDF) membrane using the iBlot 2 Dry Blotting System (Invitrogen, Waltham, MA, USA). The membrane was blocked with 5% BSA in TBS-Tween20, then incubated with primary antibody overnight at 4 °C. The membrane was washed with TBS-Tween20 and incubated with anti-mouse or anti-rabbit HRP-conjugated secondary antibody for 1 h at room temperature. The membrane was washed, and the protein was visualized using the Pierce ECL Western Blotting Substrate (Thermo Scientific, Waltham, MA, USA). Signal was visualized using Amersham ECL Prime Luminol Enhancer and Peroxide Solution (GE Healthcare, Piscataway, NJ, USA), and membranes were developed using SuperSignal™ West Pico Plus Chemiluminescent Substrate (Thermo Fisher Scientific). Membranes were exposed to film using Amersham Biosciences Hypercassettes and Denville Scientific HyBlot CL Films (Thomas Scientific, Swedesboro, NJ, USA), and film was developed in an ECOMAX™ X-ray film processor (Protec, Oberstenfeld, Germany). Band densitometry was performed using ImageJ (1.53t).

### 4.8. Immunoprecipitation

After protein quantification with a BCA assay kit, 1–2 mg of total protein cell lysate was pre-cleared by overnight addition of 40 μL of protein A/G Plus agarose beads (Santa Cruz Biotechnology, Dallas, TX, USA). The supernatant was then incubated with 2 μg of the antibody of interest at 4 °C for 6 h before overnight incubation with 40 μL of protein A/G Plus agarose beads at 4 °C under agitation. Samples were washed three times with 400 μL RIPA buffer, re-suspended in 100 μL of 2X SDS Laemmli buffer (1 mM TRIS pH 6.8, 1% SDS, 40% glycerol, 0.1% bromophenol blue, 20% β-Mercaptoethanol) and heated at 95 °C for 10 min before electrophoresis.

### 4.9. Statistical Analysis

Two-tailed Student’s *t*-tests or single-factor ANOVA tests were used to determine statistical significance in in vitro experiments. Experiments were performed at least in triplicate. 

## Data Availability

Publicly available datasets were analyzed in this study. This data can be found at the R2 Genomics Analysis and Visualization Platform (http://r2.amc.nl).

## Abbreviations

DUBs	deubiquitinating enzymes
EFS	event-free survival
EZH2	enhancer of zeste homolog 2
NB	neuroblastoma
OS	overall survival
UPS	ubiquitin proteasome system
USP7	ubiquitin specific protease 7
